# Natural history of cerebrotendinous xanthomatosis: a paediatric disease diagnosed in adulthood

**DOI:** 10.1186/s13023-016-0419-x

**Published:** 2016-04-16

**Authors:** Bertrand Degos, Yann Nadjar, Maria del Mar Amador, Foudil Lamari, Frédéric Sedel, Emmanuel Roze, Philippe Couvert, Fanny Mochel

**Affiliations:** AP-HP, Département des Maladies du Système Nerveux, Groupe Hospitalier Pitié-Salpêtrière, Paris, France; AP-HP, Département de Biochimie Métabolique, Groupe Hospitalier Pitié-Salpêtrière, Paris, France; AP-HP, UF Neurométabolique Bioclinique et Génétique, Groupe Hospitalier Pitié-Salpêtrière, Paris, France; Sorbonne Universités, GRC Neurométabolisme, UPMC Univ Paris 6, Paris, France; MedDay Pharmaceuticals, Institut du Cerveau et de la Moelle épinière, Paris, France; UPMC Univ Paris 6 UMR S 1127, Inserm U 1127, CNRS UMR 722, Institut du Cerveau et de la Moelle épinière, Sorbonne Universités, Paris, France; AP-HP, Service de Biochimie Endocrinienne et Oncologique, Groupe Hospitalier Pitié-Salpêtrière, Paris, France; AP-HP, Département de Génétique, Groupe Hospitalier Pitié-Salpêtrière, 47-83 Boulevard de l’Hôpital, 75651, Paris, Cedex 13 France

**Keywords:** Cerebrotendinous xanthomatosis, Diarrhea, Cataract, Cerebellar ataxia, Cognitive dysfunction, Psychiatric symptoms

## Abstract

Cerebrotendinous xanthomatosis (CTX) is among the few inherited neurometabolic disorders amenable to specific treatment. It is easily diagnosed using plasma cholestanol. We wished to delineate the natural history of the most common neurological and non-neurological symptoms in thirteen patients with CTX. Diarrhea almost always developed within the first year of life. Cataract and school difficulties usually occurred between 5 and 15 years of age preceding by years the onset of motor or psychiatric symptoms. The median age at diagnosis was 24.5 years old. It appears critical to raise awareness about CTX among paediatricians in order to initiate treatment before irreversible damage occurs.

**Electronic supplementary material:**

The online version of this article (doi:10.1186/s13023-016-0419-x) contains supplementary material, which is available to authorized users.

## Introduction

Cerebrotendinous xanthomatosis (CTX) is a rare autosomal recessive sterols storage disorder caused by 27-sterol-hydroxylase deficiency due to *CYP27A1* mutations resulting in an accumulation of cholestanol in blood and organs, mainly the central nervous system, eyes, tendons and vessels [1, 2]. With an estimated prevalence of 1/50,000 [3] but only about 300 patients reported, CTX remains too often under- or misdiagnosed while treatment is available. Patients typically manifest both systemic and neuropsychiatric symptoms of the disease. Systemic manifestations may include infantile cholestasis or liver dysfunction, juvenile cataract, Achilles tendon xanthomas, osteoporosis, premature arteriosclerosis and cardiovascular disease [1, 2, 4]. Neurological symptoms encompass cognitive delay, spastic paraplegia, cerebellar ataxia, peripheral neuropathy, bulbar palsy, epilepsy, movement disorders, dementia and psychiatric disturbances [1, 2, 4]. While these symptoms are well described, their natural history is not. More importantly, CTX is almost always diagnosed in adults whereas most of the initial symptoms occur in childhood and adolescence. We therefore describe the natural history of the most common neurological and non-neurological symptoms in thirteen patients with CTX in order to alert on the early symptoms of the disease.

## Patients and methods

We collected retrospectively clinical, biochemical, imaging and electrophysiological data from thirteen genetically confirmed CTX patients followed at La Pitié-Salpêtrière University Hospital. In order to delineate the natural history of the disease, we selected only patients whose diagnosis was made in adolescence or adulthood. Values are expressed in mean ± SD and/or median. For the Kaplan-Meier analyses, we assumed that patients are determined to have CTX at birth. Therefore all time to event data (e.g. time to diarrhea, time to cataract) assumed that the initial time was the date of birth. The censoring time was the age at latest neurological exam. The symptoms were communicated via the caregiver or the patient directly. Each patient gave a written informed consent to participate in the study. The study was approved by the local ethics committee (CPPIdF6, La Pitié-Salpêtrière University Hospital).

## Results

We describe four males and nine females with CTX belonging to ten families. Four patients were born from a consanguineous union (Additional file 1). All patients were Caucasian except two patients of Turkish origin. Six patients were compound heterozygous and five patients homozygous for *CYP27A1* mutations. In two siblings, the second mutation was not identified despite very high levels of plasma cholestanol (Additional file 1). In three families, the diagnosis of the index case led to the diagnosis of a sibling. The mean and median age at diagnosis were 30.4 ± 14.9 years and 24.5 years (range: 14-55) respectively. The mean and median disease duration at the time of diagnosis were 26.2 ± 11.6 years and 21.5 years (range: 13.5–54.5) respectively.

**Fig. 1:**
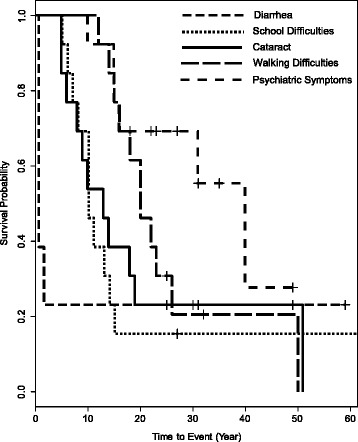
Kaplan–Meier analyses indicate the natural history of thirteen patients with CTX for time to diarrhea, cataract, school difficulties, walking difficulty and psychiatric symptoms

The natural history of our CTX cohort revealed that chronic diarrhea was very common, starting within the first year of life (Table 1, Fig. 1). None of our patients had a known history of infantile hepatic dysfunction. Cataract and school difficulties usually followed between 5 and 15 years of age (Additional file 1). Another 5–15 years later, most patients developed motor dysfunction leading to walking difficulties and/or psychiatric symptoms (Fig. 1, Additional file 1). Plasma cholestanol was markedly elevated in all patients with a mean of 64 ± 23 μmol/l (35–98 μmol/l, normal range: 2-10 μmol/l) (Additional file 1).

**Table 1:** Clinical characteristics in a cohort of thirteen patients with CTX

Demographic			
- Gender	Female: 9	Male: 4	
- Familial genetics	Consanguinity: 4	Affected sibs: 6	
Age of onset [median; mean ± SD; range] (years)			
- Diarrhea	10/13, neonatal		
- School difficulties	11/13 [10; 9.9 ± 3.2; 5–15]		
- Cataract *(age of surgery)*	11/13 [13; 15.4 ± 13.8; 5–54]		
- Psychiatric symptoms	6/13 [15.5; 21.2 ± 11.7; 10–40]		
- Walking difficulties	11/13 [20; 21.4 ± 10.3; 12–50]		
Neurological examination			
Age at examination [median; mean ± SD; range] (years)	30; 33 ± 13.8 (18–60)		
- Dysmetria	Yes: 7/13		
- Tandem	Unable: 5/13	Abnormal: 7/13	Normal: 1/13
- LL spasticity	Yes: 6/13		
- UL spasticity	Yes: 0/13		
- LL reflexes (knee)	Increased: 6/13	Absent: 3/13	Normal: 4/13
- LL reflexes (ankle)	Increased: 5/13	Absent: 3/13	Normal: 5/13
- UL reflexes	Increased: 9/13		Normal: 4/13
- Plantar reflexes	Upgoing: 7/13		Flexor/Indifferent: 6/13
- Romberg	Positive: 3/12		Negative: 9/12
- LL proprioception	Decreased: 10/11		Normal: 1/11
- UL proprioception			Normal: 11/11
Eye movements			
- Pursuit	Saccadic: 8/13		Normal: 5/13
- Saccades	Dysmetric: 7/13		Normal: 6/13
Cognitive dysfunction	13/13		
- Delayed cognition	10/13		
- Dysexecutive/Decline	12/13		
Paroxysmal manifestations			
- Myoclonic dystonia	7/13		
- Epilepsy	1/13		
Osteoporosis	4/13		
Tendon Xanthoma	3/13		
Peripheral neuropathy	10/13 - Axonal (4/10), Demyelinating (5/10), Mixed (1/10)		
Brain MRI/MRS			
- Global atrophy	3/13		
- Periventricular T2 hyperintensities	10/13		
- Increased choline peak (MRS)	13/13		
- Dentate nuclei T2 hyperintensities	12/13		
- Cerebellar atrophy	7/13

A detailed neurological evaluation was performed at a median age of 30 years (18–60). Except for two patients with no overt motor dysfunction, CTX patients presented mainly with cerebello-pyramidal (6/13), neuropathic (3/13), cerebellar (1/13) or pyramidal (1/13) dysfunction. Seven patients also displayed postural myoclonic jerks associated with dystonia, including six that were confirmed by electrophysiological recordings [5]. Cognitive functions were altered in all patients and a neuropathy was documented in ten patients (Table 1). On clinical examination, only 3/13 patients had clinical tendon xanthomas (Table 1). Four patients developed osteopenia but none experienced cardiac or pulmonary manifestations (Table 1). Typically, brain MRI showed T2-weighted hyperintensities of the dentate nuclei and brain MR spectroscopy identified an increased peak of choline in all patients (Table 1).

## Discussion

We describe the natural history of 13 patients with CTX. Chronic diarrhea was the earliest symptom commonly observed in CTX but did not seem to be a main cause for consultation, possibly due to the absence of associated growth retardation [6, 7]. Nonetheless, paediatric gastroenterologists should search for CTX in the context of an early onset diarrhea, especially with a history of infantile hepatic dysfunction. Similarly, the diagnosis of a cataract in a child or an adolescent must lead to measure plasma cholestanol. In our cohort, cataract and school difficulties tended to occur at a similar age suggesting that visual difficulties may contribute to the early cognitive difficulties of some CTX patients. Years after the onset of diarrhea, cataract and/or school difficulties, many patients developed gait abnormalities primarily related to cerebellar and/or pyramidal dysfunction and combined with cognitive dysfunction. About half of our cohort also developed psychiatric symptoms. Tendon xanthomas were rarely found on clinical examination, which emphasizes that their absence should not delay the diagnosis of CTX [6]. Electromyography often revealed a peripheral neuropathy with either an axonal or a demyelinating pattern. Brain MRI also showed T2-weighted hyperintensities of the dentate nuclei and are suggestive of the diagnosis [8]. The increased peak of choline on MR spectroscopy might be useful to monitor response to therapy.

It has been shown that non-neurological and neurological manifestations can respond to chenodeoxycholic acid (CDCA) through decreased plasma cholestanol levels [1, 4, 9]. When initiated early, the therapeutic response to CDCA can be dramatic [10, 11]. Therefore, paediatricians must be at the forefront of diagnosing CTX in children with chronic diarrhea and/or cataract and/or learning difficulties. Indeed, such symptoms constitute an important therapeutic window to initiate treatment in patients with CTX before the onset of disabling motor and psychiatric symptoms.

## Conclusion

Our work shows that CTX is almost always diagnosed in adults whereas key symptoms occur in childhood and adolescence. Treatment shall be initiated before the onset of disabling motor and psychiatric symptoms.

## Consent for publication

Not applicable (manuscript contains no individual person’s data).

## Additional file

Additional file 1: Demographic, biochemical and genetic characteristics, and age of onset of key symptoms in a cohort of thirteen patients with CTX. (XLSX 10 kb)

## Abbreviations

CDCA: chenodeoxycholic acid
CTX: cerebrotendinous xanthomatosis
